# Immune disease expressed in liver and platelets in an adolescent: a case report

**DOI:** 10.1186/1824-7288-36-42

**Published:** 2010-06-11

**Authors:** Henedina Antunes, Ruben Rocha, Nicole Silva, Teresa Pontes, Ana Antunes, Sofia Martins

**Affiliations:** 1 Adolescents Unit and Gastrenterology, Hepatology and Nutrition Unit, Pediatrics Department, Braga Hospital, Braga, Portugal; 2 Life and Health Sciences Research Institute (ICVS), School of Health Sciences, University of Minho, Campus de Gualtar, 4709-057 Braga, Portugal

## Abstract

We report a case of a 15-year-old boy with autoimmune hepatitis lacking common serologic markers and normal gammaglobulinemia associated with immune thrombocytopenia and family history of psoriasis. He presented to our department with a 4-year history of a cervical posterior lymphadenopathy and recent petechiae. Previous laboratory results 6 months before already showed hepatocellular injury. After exclusion of other causes, the diagnosis of autoimmune hepatitis was made based on clinical grounds, associated immune disorder and histological features of liver biopsy.

The authors alert for this atypical presentation of autoimmune hepatitis and associated immune thrombocytopenia.

## Background

Autoimmune hepatitis is a chronic necroinflamatory liver disorder often associated with other autoimmune diseases, like rheumatoid arthritis, Sjögren syndrome, chronic thyroiditis, ulcerative colitis, celiac disease or others. Association with immune thrombocytopenic purpura is unusual, especially in children [1]. We report a 15-year-old-boy with a family history of psoriasis, who presented with cervical lymphadenopathy, severe immune thrombocytopenia and chronic active autoimmune hepatitis successfully treated with prednisolone and azatioprine.

## Case Presentation

A 15-year-old caucasian boy presented to our emergency department with a 4-year history of a posterior left cervical lymphadenopathy and petechiae in his legs that appeared 2 days before.

Four years before the admission he noted a lump in the left posterior triangle of his neck, measuring about 1.5 cm of diameter, no tender and without inflammatory signs. At that time he had no fever or other symptoms. The cervical echography showed an ovoid shaped lymphadenopathy of 21 mm in the posterior cervical left triangle of the neck and laboratory evaluation revealed a normal blood count and normal levels of liver enzymes. The serologic testing for Epstein Barr virus yielded a positive result for past infection (IgM to the viral capsid antigen negative, IgG to the viral capsid antigen positive) as did for rubella virus (IgM antibody negative, IgG antibody positive, he had been vaccinated).

The boy kept asymptomatic, except for lymphadenopathy, and 5 months before the admission he repeated laboratory tests and cervical echography. The left posterior cervical lymphadenopathy didn't show any change in size and there was no abnormality in cells blood counts, but the chemistry panel demonstrated an elevation of serum alanine aminotransferase (256 UI/L (normal (NR) 10-34 U/L)), serum aspartate aminotransferase (123 UI/L (NR 10-44 U/L)), with normal serum alkaline phosphate (311 UI/L (NR 45-122 U/L)) and lactic dehydrogenase (318 UI/L (NR 240-480 U/L)).

Two days before admission the boy noted petechiae in his legs and after being attended by his primary care physician, he was instructed to come to our emergency department.

Past medical history was remarkable for recurrent media otitis and mastoiditis at the age of 5, followed by adenoidectomy and myringotomy with 6 years old and asthma (not medicated and without exacerbations in the last year). He had no history of drug allergies nor blood transfusion and was not taking any medications or drinking alcohol. His immunizations were up to date. His mother and three uncles have psoriasis. His social history was unremarkable.

On physical examination, the boy appeared well, in no distress and without jaundice. His vital signs were normal. No physical signs of chronic or acute liver disease were found, and the remaining examination was normal, except for petechiae in his legs and an ovoid shaped cervical posterior lymphadenopathy of approximately 2 cm in diameter, rubbery consistency, no tender and without external inflammatory signs. No other lymphadenopathy or hepatosplenomegaly were found.

Laboratory results included: hemoglobin 14.5 g/dL, white blood cell count 7.60 × 10^9^/L (42.0% neutrophils, 2.4% eosinophils (182/μL)), platelet count 6 × 10^9^/L, C reactive protein 0.34 mg/L, aspartate aminotransferase 820 UI/L (NR 10-44 U/L), alanine aminotransferase 1568 UI/L (NR 10-34 U/L), gamma glutamyl transferase 84 UI/L (NR 11-50 U/L), alkaline phosphate 223 UI/L (NR 45-122 U/L), total bilirubin 1.10 mg/dL (NR 0.2-1.0 mg/dL), unconjugated bilirubin 0.17 mg/dL (NR < 0.3 mg/dL), activated partial thromboplastin time 35.7 s (NR 25.0-35.0s) prothrombin time 15.4s (NR 8.0-14.0) (International Normalized Ratio 1.30), albumin 4.5 g/dL (NR 3.5-5.2 g/dL), immunoglobulin G 1240 mg/dL(NR < 1550 mg/dL), ferritin 1230 ng/mL (NR 20-300 ng/mL). He was heterozygous for the H63D mutation of the HFE gene. Ceruplasmin, copper and alpha-1-antitrypsin levels were normal. Viral serologies: Hepatitis A antibody IgM and IgG negative, Hepatitis B surface antigen negative, Hepatitis B core antibody negative, Hepatitis B e antigen and antibody negatives, Hepatitis B surface antibody negative, Hepatitis C antibody negative and HIV I/II antibodies negatives. Epstein Barr Virus (IgM to the viral capsid antigen negative, IgG to the viral capsid antigen positive, IgM to the early antigen negative, and antibody to EBNA positive) and Cytomegalovirus (CMV IgM antibody negative, CMV IgG positive) serologic results showed past infection. Testing for antinuclear, antimitochondrial, anti-smooth muscle, and liver/kidney microssomal antibodies were negative. Abdominal ultrasound showed a normal homogeneous echo texture of liver and a normal spleen size of 11 cm.

Liver biopsy was done when the patient had 63 × 10^9^/L platelets (5 days after admission), and revealed an inflammatory lymphocytic infiltrate in portal areas with eosinophils. Some areas showed interface hepatitis ("piecemeal necrosis") with inflammatory lymphocytic infiltrate eroding the limiting plate of hepatocytes and bridging fibrosis with porto-portal fibrous septa and fibrous in the peri-portal area. Bile ducts were normal.

Blood smear, bone marrow aspirate and flow cytometry of peripheral blood cells were negative for neoplasic cells and pointed to peripheral destruction of platelets cells with increased number of megakaryocytes in bone marrow and rare but big platelets in blood smear. Anti-platelet antibodies, detected by flow cytometry, were positive: IgG 4.32 AU(immunofluorescence arbitrary unit, normal < 0.33 AU); IgM 0.39 UA (normal < 0.29 AU).

Based on all results we establish the diagnosis of immune thrombocytopenia and autoimmune hepatitis with negative serologic markers.

The patient was initially treated with intravenous immunoglobulin for thrombocytopenia with a partial response and then with prednisolone. He started azathioprine after the diagnosis of autoimmune hepatitis.

He has been followed by about two years and has been medicated with azathioprine (1.4 mg/kg/day). After 2 months of the admission, platelets count recovered to 96 × 10^9^/L and lymphadenopathy decreased and disappeared in the next months. After 10 months platelet count was normal 158 × 10^9^/L. Liver enzymes returned to normal after 2 months, aspartate aminotransferase 15 UI/L (NR 10-44 U/L), alanine aminotransferase 18 UI/L (NR 10-34 U/L), gamma glutamyl transferase 44 UI/L (NR 11-50 U/L).

## Discussion

Autoimmune hepatitis (AIH) is a chronic and progressive necroinflammatory and fibrotic process of the liver of unknown cause that occurs in children and adults, usually associated with the presence of autoantibodies and hypergammaglobulinemia[2].

It has been described two main types of autoimmune hepatitis based on differences in autoantibody patterns. AIH type I is characterized by the presence of circulating anti-smooth muscle antibodies (SMA) and/or antinuclear antibodies (ANA) and AIH type II is characterized by circulating liver-kidney microssomal type I antibody (LKM) or anti-liver cytosol antibody (LCA) [2,3]. Eighty percent of patients with AIH type II are children.

The diagnosis is made regarding clinical and biochemical findings of hepatitis and associated autoimmune diseases, abnormal levels of immunoglobulin G, the presence of the characteristic autoimmune antibodies and finally, the most important, the histological typical abnormalities [2,4].

The International Autoimmune Hepatitis Group proposed a scoring system to standardize the diagnosis, useful for clinical trials, but that may be inaccurate in individual cases, especially in children [2,5].

After excluding viral, toxic and hereditary aetiologies for hepatitis, in the presence of another autoimmune disease and considering liver biopsy characteristics, the most probable cause for hepatitis in our patient was certainly an autoimmune process. Nevertheless, we didn't find the common serologic markers of this disease (SMA, ANA, LKM and LCA), neither characteristic hypergammaglobulinemia, nor meet the criteria for definitive diagnosis for autoimmune hepatitis according to the International Autoimmune Hepatitis Group criteria or the simplified criteria for the diagnosis of autoimmune hepatitis published in 2008 by Hennes EM *et al *[6].

According with the International Autoimmune Hepatitis Group criteria for diagnosis of autoimmune hepatitis[5], the patient scored 13 points before treatment and didn't fulfil the criteria for a definitive diagnosis of autoimmune hepatitis (it's important to note that we didn't test for genetic markers like HLA DR3 and HLA DR4, which positivity could mean one point more in the score, but not enough to fulfil the criteria).

Autoantibodies may be absent in about 10% of patients with autoimmune hepatitis and in children the presence of autoantibody titers tends to be lower, and may be not detected, as in this case[2,7]. They serve as markers of disease, but their pathogenic role and their value for follow-up evaluation remains controversial [8].

Petechiae (thrombocytopenia) were the major complain that brought the patient to medical attention. Based on blood smear, bone marrow aspirate and positive anti-platelets antibodies we presumed an immune destruction of platelets and assumed the diagnosis of immune thrombocytopenia.

Idiopathic thrombocytopenia purpura is a well recognized complication of viral hepatitis, but the association between autoimmune hepatitis and immune thrombocytopenic purpura is not frequent, especially in children [1]. There are some case reports in literature showing this association, but almost all were in adults [1,9-15]. Wong T (2007) presented a case of a 10-year-old boy with autoimmune hepatitis and a 6 month history of gingival bleeding and epistaxis due to coagulopathy, with elevated prothrombin time and partial thromboplastin time but normal platelets count [11]. Our boy had also a prolonged INR, reverted by K vitamin, but no clinical manifestations of bleeding.

Shibuya A (2001) presented a 19 year-old woman with typical AIH, positive serologic markers and fulfilment of diagnostic criteria score and thrombocytopenia. Four years later the woman developed thrombotic thrombocytopenic purpura and died because of multi-organic failure 9 days after the admission [9].

In 1996, Persico M described thrombocytopenia as a sensitive marker of immunologic activity in a patient with autoimmune chronic active hepatitis [16]. This case is a good demonstration of it. The appearance of thrombocytopenia was correlated with the increasing activity of hepatic lesion pointing to the existence of a single pathophysiologic process. What triggers the necroinflammatory process of the liver, the exacerbation of the hepatic lesion and the immune destruction of platelets remains obscure.

Lymphadenopathy found in patient was probably a casual association or a sequel of a past infection. Follow up confirmed its disappearance.

In conclusion, the authors alert for the infrequent presentation and associations of this case. First, presentation was atypical as it occurs in a male patient, (autoimmune hepatitis is much more frequent in females) and pethechiae were the sign that brought the patient to medical attention and led to the diagnosis. Furthermore, the patient didn't have the typical serologic markers of autoimmune hepatitis, neither the commonly associated hypergammaglobulinemia or fulfil the diagnostic criteria for definitive diagnosis. Second, immune thrombocytopenia is not a common autoimmune disease associated to autoimmune hepatitis and family history revealed a strong presence of psoriasis (another autoimmune disease).

## Consent

Written informed consent was obtained from the patient for publication of this case report. A copy of the written consent is available for review by the Editor-in-Chief of this journal.

## Abbreviations

AIH: Autoimmune hepatitis; SMA: Anti-smooth muscle antibodies; ANA: Antinuclear antibodies; LKM: Liver-kidney microssomal type I antibody; LCA: Anti-liver cytosol antibody;

## Competing interests

The authors declare that they have no competing interests.

## Contributor's list

HA had primary responsibility in treating the patient and manuscript review.

RR participated in patient treatment and was responsible for writing the manuscript.

NS participated in patient evaluation and treatment.

TP contributed in the same way as Nicole Silva.

AA contributed in the same way as Nicole Silva.

SM participated in patient treatment and was responsible for manuscript review.

All authors have read and approved the final manuscript.
